# The Organised Theft of Medicines: a Study of the Methods for Stealing and Reselling Medicines and Medical Devices in the EU and Beyond

**DOI:** 10.1007/s10610-023-09546-w

**Published:** 2023-05-31

**Authors:** Marco Dugato, Cosimo Sidoti

**Affiliations:** Università Cattolica del Sacro Cuore - Transcrime, Largo Gemelli, 1–20123, Milan, Italy

**Keywords:** Theft of medicines, Pharmaceutical crime, Organised crime, Illicit markets, Crime script analysis

## Abstract

The theft of medicines is a significant component of the illicit trade in pharmaceutical products. Besides small-scale thefts committed for personal usage, organised criminal networks are increasingly targeting high-priced medical products, either to reintroduce them into the legal supply chain or sell them on the black market. This crime has considerable implications that extend beyond the value of the stolen goods, including harmful impacts on citizens’ health, legitimate companies, and national health systems. However, knowledge on organised theft of medicines remains limited. This paper employs a crime script analysis approach, based on interviews with relevant stakeholders and case studies retrieved across European countries, to examine the most common *modi operandi* in the organised theft of medicines and medical devices. Potential policy implications are also discussed.

**Supplementary Information:**

The online version contains supplementary material available at 10.1007/s10610-023-09546-w.

## Introduction

Organised theft of medicines refers to the systematic illegal taking of medicines or medical devices with the intention of reintroducing the stolen products into the legal supply chain or selling them on the black market for financial gain. It is a planned and profit-oriented activity, unlike small-scale thefts committed for personal use by criminals or their acquaintances. Although the organised theft of medicines lacks an official legal definition by competent authorities, it can be broadly comprised by Article 8 of the Medicrime Convention within the illicit trafficking of pharmaceuticals other than counterfeiting (Council of Europe, 2011)^1^ and by Interpol in their definition of pharmaceutical crime (Interpol, 2013).^2^

Criminals engage in such criminal activity for a variety of reasons. These include the increasing demand for medicines and medical devices for both licit (OECD, 2021; Vander Beken, 2007) and illegal purposes (Di Giorgio, 2022; Savona et al., 2018); the uneven accessibility or availability of these products in the legal market (Musazzi et al., 2020; Riccardi et al., 2014; UNODC, 2022); the existing asymmetries in prices and reimbursement regimes (Brasola et al., 2018); the complexity and fragmentation of the legitimate distribution network for pharmaceuticals (AIFA, 2014a; Ekwall et al., 2016; Savona et al., 2018; Zabyelina & Noguera, 2022); the growing percentage of online sales that allow criminals to sell stolen products in a more secure way (Lavorgna, 2015; Zabyelina & Noguera, 2022); and the light penalties for the theft and trafficking of medicines within the current legislation of many countries, which make this crime a low-risk activity in comparison to other illicit markets (Hall et al., 2017; OECD & EUIPO, 2020; Savona et al., 2018).

The organised theft of medicines generates a series of impacts that go beyond the monetary value of the stolen products (Shepherd, 2015). This type of crime can have severe impacts on public health, legitimate companies, and national healthcare systems (Savona et al., 2018). On the one hand, the stolen products are not subjected to any quality standards related to storage and transportation (e.g. maintaining an adequate temperature or avoiding contamination by other products). Thus, the individuals who use them may not effectively treat their medical needs or even cause further damage to themselves (Riccardi et al., 2015; Venhuis et al., 2018; Zabyelina & Noguera, 2022). Consequently, the theft of medicines can impact mortality rates, increase the prevalence of diseases, and decrease public trust in the healthcare system (OECD & EUIPO, 2020; WHO, 2017). On the other hand, it affects manufacturers, legitimate traders, and transportation companies who lose their reputation and profits due to both thefts and the associated costs of prevention, detection, and recovery (Riccardi et al., 2015; WHO, 2017). This type of crime also results in increased regulatory and enforcement expenses and higher healthcare costs, affecting national governments and healthcare systems (OECD & EUIPO, 2020). Moreover, the theft of medicines is often a transnational crime that not only affects the countries where the thefts occur but also those where the stolen medicines are laundered or intended for sale (Di Giorgio, 2020).

Despite its relevance, the organised theft of medicines remains an understudied crime (Di Giorgio, 2022). First, there is a lack of comparable and focused data on this crime, which prevents a comprehensive understanding of the issue. The few available databases that attempt to collect information on the theft of medicines are mainly managed by private entities, such as associations like the Pharmaceutical Security Institute (PSI) or the Transportation Asset Protection Association (TAPA). The available records are provided voluntarily by their members; this likely generates biases due to their individual willingness and awareness to report all incidents. In addition, pharmaceutical thefts are also either largely not reported to the authorities or not properly classified by them because of the absence of a shared definition, the lack of proper systems to classify these events, and the low level of awareness of many actors in the supply chain (Shepherd, 2017). Evidence of unreported theft of medicines can also be found in the significant number of cases of “lost” medicines recorded within healthcare facilities or during shipments. These losses may correspond to actual thefts, especially when they involve expensive or appealing products (Di Giorgio, 2020). These considerations entail that the available quantitative data should be considered partial and thus only indicative of the issue as a whole. Second, most of the information currently available belongs to judicial cases and operations that occurred several years ago, such as the Volcano Operation in 2014–2015 (AIFA, 2014b). This investigation carried out by Italian authorities was pivotal as it showed for the first time the transnational nature of the organised theft of medicines (Di Giorgio, 2015). However, there is the need to assess the current validity and the extent of the *modi operandi* depicted in those investigations. Third, most of the political and research attention on pharmaceutical crimes has largely focused on counterfeiting (Riccardi et al., 2014), despite recent evidence indicating more cases of illegal diversion^3^ of legitimate pharmaceutical products than of falsification (Di Giorgio & Russo, 2019). For example, according to the data collected by PSI, cases of illegal diversion are on the rise and account for 72% of the reported criminal events related to pharmaceutical products in 2020 (PSI, 2021). Given that the identified diverted products are likely to originate from some form of theft or fraud, the increased relevance of these diversion cases may hide the attendant increase in cases of theft of medicines.

These considerations serve to underscore the reasons why an increased awareness about this crime and its contrast must be a priority for both public authorities and private stakeholders in the pharmaceutical sector. This paper aims to contribute to the current knowledge on the organised theft of medicines and medical devices by providing a comprehensive overview of the main criminal schemes and *modi operandi* used by criminals to steal and resale medicines and medical devices across European Union member states (EU MS).

## Methodology and Data

The organised theft of medicines is a complex crime. To cope with this complexity and delineate a typology of the criminal schemes adopted by criminals, this study conducted a crime-script analysis (CSA). The CSA approach focuses on investigating the different stages of a crime (Cornish, 1994). This approach has been applied successfully to the analysis of both property crimes and organised criminal activities (Brayley et al., 2011; Dehghanniri & Borrion, 2021; Kennedy et al., 2018; Lavorgna, 2015; Savona, 2010; Savona & Riccardi, 2018). Re-elaborating the original concept proposed by Cornish (1994), the theft of medicines was deemed to include three different stages which are both logically and consequentially interconnected: pre-conditions, the theft, and post-conditions. Each of these stages can be further subdivided into specific components or elements. In specific, the pre-conditions stage relates to the management and organisation of the actors involved in the commission of the theft and the selection of the specific pharmaceutical products that get stolen; the theft stage refers to the chain of actions that lead to the theft of medicines, including selecting and accessing the places where the products are located and the methods employed to steal them; and the post-conditions stage pertains to the aftermath of the theft commission, focusing specifically on the transportation and storage of stolen products, the selling channels used by criminals, and the types of clients who finally purchase the products.

To identify the different modalities of these stages and the consequent *modi operandi* underlying the theft of medicines, the study collected data and information from a variety of sources. These include interviews with representatives from different relevant public and private entities and the analysis of case studies retrieved from judicial documents or news. In specific, a total of 15 in-depth online interviews were conducted including representatives of police forces, prosecutors’ offices, health regulatory authorities, hospital pharmacists or end-vendors, international organisations, pharmaceutical companies, transportation companies, and parallel traders. The interviews were carried out during the months of February and March 2022, and the respondents were based in eight different countries: Italy (*n* = 6), Belgium (*n* = 2), the Netherlands (*n* = 2), France (*n* = 1), Germany (*n* = 1), Serbia (*n* = 1), Sweden (*n* = 1), and Switzerland (*n* = 1). However, due to their roles or their affiliation with international authorities or multinational companies, most of them had a broad understanding of events occurring in multiple countries within the EU and beyond (see Table 1 in the Appendix). The respondents were all anonymised to protect their identity as agreed in the informed consent. Furthermore, a set of case studies was obtained by analysing documents related to six judicial cases that involved multiple countries within and beyond the EU, providing evidence of the transnational nature of this crime (see Table 2 in the Appendix). Additionally, data on 87 incidents of thefts of medicines were collected by systematically scraping news and press releases from across EU MS between 2015 and 2022 (April).^4^ Sixteen different EU MS or geographically close non-EU countries were mentioned within these 87 news stories (see Table 3 in the Appendix). The data collection focused on European cases, but anecdotal evidence and considerations emerging from the interviews suggest that the identified cases are also representative of other contexts worldwide.

The study analysed the available evidence related to cases of the theft of medicines or medical devices using the CSA approach to classify them as recurrent criminal schemes. The collected information was manually coded and sub-coded according to the pre-established stages and specific components of the theft of medicines. A criminal scheme refers to a general pattern that represents a group of similar cases where criminals use a comparable *modus operandi*. However, it must be reminded that criminal schemes should not be considered mutually exclusive, but rather as a concatenated flow of actions that can also co-exist and be performed simultaneously. Likewise, the same criminal actors may be involved in multiple schemes and interchange their functions.

## Criminal Schemes

The following sections provide an overview of the seven criminal schemes that were identified.^5^

### Scheme 1: International Illicit Traders

The objective of this scheme is to steal large amounts of expensive medicines and reintroduce them into the legal supply chains of other countries using falsified documents. This illicit activity is primarily carried out by organised criminal networks who exploit their connections in both legal and illegal markets. On the one hand, they may hire local gangs or corrupt employees in hospitals, warehouses, and transportation couriers. On the other hand, they connect with conniving pharmaceutical wholesalers or professionals to launder and resell the stolen products (Hospital pharmacists, personal communication, February 15, 2022). Medicines are stolen along all the various stages of the supply chain, including production facilities, cargos, warehouses, or hospitals. The stolen medicines are then transported and stored in clandestine illegal warehouses before being inventoried and laundered. This often involves the creation of shell companies abroad that provide false documentation. The laundered medicines are reintroduced into the pharmaceutical legal supply chain mainly through parallel trading, facilitated by complicit or bogus wholesalers (Di Giorgio, 2015). Legitimate parallel exporters—who are either unaware or financially motivated—legally purchase the illicit products from these wholesalers and export them to importing countries. The final destinations of these medicines are hospitals or other legitimate medical premises, like private health clinics, that purchase them usually unwittingly (Prosecutor, personal communication, February 23, 2022). This criminal scheme was a profitable business for many years in the EU, particularly from 2014 to 2018 (Di Giorgio, 2020). However, it is now declining due to increased hospital security measures (Hospital pharmacists, personal communication, February 15, 2022) and the implementation of better medicine traceability systems across EU MS, which have reduced opportunities for laundering and reselling stolen medicines (Parallel Trader, personal communication, February 15, 2022).

### Scheme 2: Suppliers

Organised criminals operating across borders lead a scheme that involves stealing small quantities of high-priced medicines, which are then exported to countries where these pharmaceuticals are either unavailable or not covered by national health systems. Alternatively, the stolen products may be sold within the same country to patients who are willing to pay out-of-pocket expenses. Criminals rely on existing connections or community ties to coordinate different functional units (Ministero della Salute, 2019; Tribunale di Cremona, 2018). The thefts are commissioned to specialised gangs who are tasked with stealing the required products (Police Officer, personal communication, February 7, 2022). Specialised gangs are commissioned by healthcare facilities in the destination countries or by professionals acting as brokers between thieves and patients. Medicines are primarily stolen from hospital pharmacies, but warehouses and production facilities can also be targeted. The stolen medicines are stored in proprietary illegal warehouses or facilities and then transported abroad through personal luggage (also known as “ant-smuggling”) or through postal services disguised as legitimate deliveries to companies or individuals. The preferred clients for these stolen products are wealthy individuals or private medical premises, such as health clinics or private doctors (Police Officer, personal communication, February 7, 2022).

### Scheme 3: Generalists

The aim of criminals is to steal medium- to large-sized quantities of mainly over the counter (OTC) medicines or generic medical devices with the intention of furnishing local pharmacies or private medical facilities. The main players in this scheme are professionals in the legal pharmaceutical market, such as pharmacists, doctors, or wholesalers (Di Giorgio & Russo, 2019). These professionals leverage their knowledge and connections in the legitimate market to launder and sell stolen products while enlisting local criminals to carry out the thefts (Prosecutor, personal communication, February 23, 2022). These thieves are not necessarily specialists in stealing medicines, but they are often involved in a wide range of criminal activities. Medicines are stolen from cargos, hospitals, or pharmacies and are stored in illegal warehouses owned by the criminals. The stolen products are then sold through fake online pharmacies and marketplaces or reintroduced into the legal supply chain by falsifying transaction documents to make them appear legitimate (Prosecutor, personal communication, February 23, 2022). The final destinations are unwitting local pharmacies, healthcare premises owned or operated by the criminals, or individuals through online channels (Health Regulatory Authority 1, personal communication, February 22, 2022).

### Scheme 4: Recyclers

The objective of this scheme is to steal waste or expired medicines, as well as small quantities of new pharmaceuticals, in order to supply counterfeiters with samples or materials of new or highly demanded products. Criminal groups involved in the counterfeiting of medical products are the main actors in this scheme and usually commission the thefts to local gangs or thieves. The latter take advantage of the opportunities created by the vulnerabilities in hospitals, pharmacies, or other healthcare facilities to commit thefts, which may include stealing from garbage (Ruggeri, 2020). Although less frequent, thefts from cargos or production sites may occur through small-scale burglaries or thefts supported or perpetrated by conniving employees. Once stolen, the products are then provided to criminals who are specialised in the falsification and adulteration of medicines. These criminals also rely on conniving professionals in the pharmaceutical sector to identify the types of products that are in higher demand and how best to retrieve them (Pharmaceutical Security Manager 3, personal communication, February 25, 2022).

### Scheme 5: Dealers

The aim of these criminals is to steal medical products that can be used for illicit or recreational purposes and sell them on the black market (Di Giorgio, 2020). The management of this criminal scheme consists of decentralised entities (Antonopoulos & Hall, 2016), and the main protagonists are brokers primarily from the fitness and sporting sectors (e.g. gym managers or owners, bodybuilding instructors, staff members of sports teams and federations), animal sports (e.g. breeders, veterinarians, and drivers), or sex-related services and adult entertainment (e.g. adult movie actors or producers, managers of escort agencies, sex shop owners) (Paoli & Donati, 2014; Policía Nacional, 2016). Most of these actors have no prior criminal records, have legitimate professions, and are in direct contact with their clients, often sharing the same environment. As such, they often operate as retailers, selling the stolen products by exploiting their legitimate professional affiliations and relationships (Health Regulatory Authority 1, personal communication, February 22, 2022). Medicines are stolen from a wide range of sources including cargos, warehouses, hospitals, and pharmacies. Healthcare employees and physicians are often involved in the theft. The stolen medicines are then transported and stored in illegal warehouses or conniving businesses. Unlicensed online pharmacies or marketplaces are the primary selling channels, but medicines are also sold directly to individuals via face-to-face interactions often within conniving or unwitting legal businesses (e.g. gyms and sex shops). The destination of the stolen products is often local, but the growth in online sales provides new business opportunities for criminals by giving them access to other markets nationally or abroad (Parallel Trader, personal communication, February 15, 2022).

### Scheme 6: Technicians

The objective of this criminal scheme is to steal expensive and lightweight medical equipment, such as endoscopes, surgical drills, and optical medical devices, and sell them either on the secondary or black market abroad. Small-scale thefts, usually, are carried out by specialised groups of criminals in hospitals, healthcare facilities, specialised shops, or private doctors’ offices. The thieves gain access by breaking and entering the premises or by infiltrating healthcare facilities with the support of internal personnel (Police Officer 2, personal communication, February 18, 2022).

Other operative units are involved in transporting small quantities of the stolen medical devices to the destination countries in personal luggage or using private means of transport. Alternatively, they may outsource the transportation of the stolen products to conniving transport couriers or postal services. To launder and sell these stolen medical devices, criminals collaborate with healthcare professionals to create fictitious or conniving companies. These professionals provide also valuable knowledge on how to handle these delicate devices, which can easily break and become unusable and unsellable (Police Officer 1, personal communication, February 7, 2022). The stolen products are sold at competitive prices to legitimate healthcare facilities on the black market or as reusable items on the secondary market using fake documentation (Police Officer 1, personal communication, February 7, 2022).

### Scheme 7: Merchants

Criminals aim to steal large amounts of low-priced and small-size medical devices or personal protective equipment (PPE) (e.g. facemasks, ventilators) that are in broad demand among the public and then sell them either on the black market or to unsuspecting legal businesses. This scheme is mainly carried out by small criminal groups who take advantage of opportunities through their local connections and activities within their territories (Vizoso, 2020). These groups are usually associated with conniving employees in courier services, as well as professionals in the import/export and healthcare sectors. While the former help with the commission of the crimes or provide logistic services for transporting the stolen products, the latter manage or support the sale of these items (Transport Security Manager 2, personal communication, February 16, 2022). Criminals steal from cargos during transportation or products stored in warehouses. There is frequent collusion with internal employees (Police Officer 2, personal communication, February 18, 2022). After stealing the medical devices, the thieves store them in proprietary illegal warehouses. The selling channels vary depending on the stolen quantity and management capacities of the criminal groups. They include selling the products on the black market or reintroducing them into the legal supply chain using fake documents provided by conniving professionals or bogus companies. In the case of laundering, the products are sold to unsuspecting healthcare premises or other legitimate businesses (e.g. pharmacies, convenience shops). The sales on the black market target citizens directly through conniving legitimate businesses, street vendors, or online channels such as social media, marketplaces, or dedicated websites (Pharmaceutical Security Manager 2, personal communication, February 22, 2022). This criminal scheme has been on the rise in recent years, especially during the COVID-19 pandemic, which has increased both the demand for and circulation of medical devices and PPE (Transport Security Manager 2, personal communication, February 16, 2022).

## Main Traits of the Organised Theft of Medicine

This section discusses the similarities and variations characterising the different stages of the theft of medicines as a result of a comparative analysis of the identified criminal schemes.

### Pre-conditions: Actors and Management

The theft of medicines requires an organised group of criminals with peculiar competencies and abilities to perform specific tasks. All the criminal schemes testify to how this crime largely relies on different functional units that are coordinated by single individuals or groups, who oversee by brokering or coordinating the various activities. These units are often strongly bonded by mutual trust and, in a few cases, by cultural or kinship ties (Police Officer 1, personal communication, February 7, 2022). For example, they may take advantage of ethnic ties across communities in both origin and destination countries or of already existing flows of illicit products across countries to facilitate the movements of the stolen items across borders. The level of complexity of the criminal associations varies according to the specific characteristics of the criminal schemes, such as the quantity and type of stolen items, the economic profits generated, the possible cross-border nature of the trafficking, and the presence of a pre-existing organised structure.

The different functional units can involve thieves, transporters, fences, or retailers, depending on the activities they are involved in. For example, criminal groups dealing with generic cargo crimes are typically hired or involved in the theft of the products (Police Officer 1, personal communication, February 7, 2022; Pharmaceutical Security Manager 3, personal communication, February 25, 2022). Similarly, individuals operating in other illicit trading channels (e.g. smuggling drugs or other illicit products) can be involved to move stolen medicines or equipment across borders. Finally, conniving professionals or experts in the healthcare sector are crucial to the laundering and reselling of stolen products. Based on their expertise, they can establish whitewash mechanisms and act as brokers between criminals and clients in order to sell the stolen items (Police Officer 1, personal communication, February 7, 2022; Prosecutor, personal communication, February 23, 2022). In some instances, these actors also help to either set up bogus companies or mediate with conniving ones. Conniving professionals are also crucial to provide criminals with insight into the pharmaceutical supply chain as well as how to evolve their *modi operandi* in response to the countermeasures from both public authorities and other legitimate actors. The functional units forming these criminal organisations are extremely market oriented and loosely linked among them since their cooperation is of opportunistic nature. Often the same units provide their services in different criminal networks.

### Pre-conditions: Products

Criminals can target five different types of pharmaceutical products.

#### Hospital Prescription Medicines

These medicines are administrated and marketed exclusively within hospitals. They are usually the most appealing target for criminals due to their high value, although they are also thoroughly tracked by national healthcare systems. Therefore, more sophisticated criminal schemes are required for them to be resold.

#### Other Prescription Medicines

These medicines can also be obtained only through a prescription from physicians or other licensed medical professionals, but they are usually dispensed in pharmacies. They are less remunerative than hospital prescription medicines, although some of them can reach significant values. They include most of the pharmaceuticals that are misused for illicit or recreational purposes (e.g. doping, performance enhancement, psychoactive effects). These medicines usually include specific products known as lifestyle or image and performance-enhancing drugs (IPEDs) (Di Giorgio, 2022; Turnock, 2020) such as anabolic steroids, EPO, insulin, stimulants, weight loss products, and medicines for erectile dysfunction. Although IPEDs are not much profitable, their demand is high in particular environments (e.g. professional bodybuilding, animal racing, sports, adult entertainment).

#### OTC Medicines

OTC medicines are directly sold to a consumer without a prescription from a healthcare professional. While they have lower costs compared to prescription drugs, these medicines appeal to criminals because they are subject to fewer monitoring systems by authorities. Furthermore, they have an easier resale market given the broad demand for them among the population and that most of them can be sold directly to clients in legal businesses (e.g. pharmacies, convenience shops).

#### Medical Devices or Equipment

The fourth type of pharmaceutical product falls within the category of medical devices and equipment intended for medical purposes, including PPE. These products are often even more attractive to criminals than medicines because there is less awareness and fewer safety measures related to these products. Criminals do not target all kinds of medical devices and equipment, but prefer those that are small and light, and, as such, more easily stealable and transportable. For example, in 2017, a police operation carried out by German authorities unveil a Colombian criminal organisation responsible for several thefts of endoscopes from hospitals across different EU MS (Stafford, 2017). The stolen endoscopes were then moved to Colombia either by airplane inside personal luggage or via regular delivery services. On arrival in Colombia, the products were reintroduced into the legal supply market as second-hand items coming from shell companies established in the USA, before then being sold to hospitals, private health clinics, and private doctors in several countries in America, Africa, and Europe (Police Officer 1, personal communication, February 7, 2022).

The COVID-19 pandemic has increased the criminal attractivity of some medical devices or equipment. In April 2020, the Galician Autonomous Police were notified of the theft of medical devices within a warehouse in Santiago de Compostela in Spain. Nearly two million masks were stolen, along with other medical supplies, with an estimated value of €5 million. The thieves aimed at exploiting the market shortages; this was highlighted by the fact that they only stole the materials that were most in demand at that juncture. The offenders sold the repackaged stolen goods to a wholesaler in Portugal who used his company’s logistic services to transport them into the country and managed the laundering and sale to clients in the healthcare sector (Vizoso, 2020).

A specific type of theft involves stealing radiotherapy equipment from hospitals or other healthcare facilities. These devices contain radioactive components that are attractive to terrorist groups or other criminals because they can be used to create “dirty bombs” (Menon & Kumar, 2019; Rimpler-Schmid et al., 2021). Usually, the products are kept under constant surveillance and stored with high-security measures due to the risk of contamination. Therefore, it is likely that these thefts are enabled by the complicity of employees or the targeting of abandoned or closed premises and medical waste. Although evidence of this type of theft is rare at present (Heylin, 2019), the threat should not be underestimated. For example, in 2013, a group of English nuclear experts reported that the country had experienced several incidents of missing radioactive materials from different premises, including hospitals, over the previous decade (Macalister & Halpin, 2013).

#### Pharmaceutical Waste

The fifth type of products that is subjected to theft is pharmaceutical waste. These products are no longer usable for medicinal purposes for a variety of reasons, including expiration date and non-compliance with temperature requirements. However, they are highly appealing to counterfeiters, who can either extract the active principles or re-use the packaging of the medicines (Pharmaceutical Security Manager 2, personal communication, February 22, 2022).

### The Theft: Places Where the Thefts Occur

The identified schemes testify that criminals can steal medicines or medical devices from a wide range of locations, such as hospitals, private healthcare facilities, warehouses, production sites, pharmacies, or during transport. The selection of the place of theft is largely connected to both the type and quantities of products being targeted by the criminals. For example, hospital and healthcare facilities are targeted for hospital prescription medicines and specialised equipment, while pharmacies are targeted for OTC or more generic products. The analysis also demonstrates how within the same criminal scheme thieves can target alternative locations or places mainly based on opportunistic reasons.

#### Hospitals, Healthcare Facilities, and Pharmacies

Hospitals, healthcare facilities, and pharmacies are heavily targeted because these premises are often vulnerable due to their organisational structure and lack of awareness of the risks associated with thefts (Pharmaceutical Security Manager 1, personal communication, February 9, 2022; Police Officer 1, personal communication, February 7, 2022). Both the high turnover of workers, patients, and their relatives and the frequently inadequate measures to secure the premises (i.e. alarms and surveillance) maximise the opportunities for theft and minimise criminals’ risks of being caught. Healthcare employees often contribute to the thefts, while doctors may purposely divert the products by stealing or falsifying prescriptions (Hospital pharmacists, personal communication, February 15, 2022).

#### Warehouses and Production Sites

Warehouses and production sites are also targeted because of the large quantities of pharmaceutical products stocked inside. The fact that their levels of security are significantly higher than hospitals’ means that criminals require greater organisation and better planning to illegally enter these locations (Pharmaceutical Security Manager 1, personal communication, February 9, 2022; Pharmaceutical Security Manager 3, personal communication, February 25, 2022).

#### During Transportation

Thieves can also steal pharmaceutical products during transportation, both with respect to primary and secondary distribution. Primary distribution involves the transportation from vendor supply points (i.e. production sites) to distribution centres (DCs), while secondary distribution pertains to transportation from DCs directly to the final retail points (e.g. hospitals or pharmacies). On the one hand, the former contains large volumes of products. Despite being appropriately protected against the risk of theft, primary distribution still suffers from several inherent vulnerabilities, namely frequent driver breaks at rest stops and service parking areas as well as the possibility of being attacked on highways. On the other hand, secondary distribution is characterised by shorter journeys and smaller volumes of products. This type of distribution is particularly vulnerable as it uses less secure vehicles and fewer alarm systems to prevent thefts during such transportation of medicines (Pharmaceutical Security Manager 1, personal communication, February 9, 2022; Pharmaceutical Security Manager 4, personal communication, February 25, 2022). Furthermore, secondary distribution is often sub-contracted to small local companies that may both be more easily infiltrated by criminals and not subjected to accurate due diligence controls (Transport Security Manager 2, personal communication, February 16, 2022).

### The Theft: Methods of Stealing

There are four methods of stealing in the theft of medicines that are transversal to all the criminal schemes.

#### Burglary and Theft

Burglary and theft are the most common methods used by criminals to steal pharmaceutical products. The breaking and entering physical premises ordinarily takes place at night or during the weekends because there are fewer employees and patients in the premises (Hospital pharmacists, personal communication, February 15, 2022). This activity is often well prepared and organised prior to the theft, often because there is a list drawn up of the products that need to be stolen and the inner mapping of the facilities has been provided by conniving employees (Transport Security Manager 2, personal communication, February 16, 2022). Thieves can enter the sites by breaking doors, windows, or walls, although in some cases they simply enter through unattended or unlocked entrances (Pharmaceutical Security Manager 2, personal communication, February 22, 2022). Criminals can also access hospitals or other healthcare facilities by pretending to be employees, patients, or relatives of patients before then stealing unattended medicines or medical devices (Pharmaceutical Security Manager 2, personal communication, February 22, 2022). Furthermore, the thefts may also be committed by insiders themselves, such as doctors, pharmacists, or employees within logistics companies, who take advantage of their positions to steal medicines and medical devices.

Large-scale cargo thefts during primary distribution occur either *en route* or in parking and rest areas. Thieves either cut the tarpaulin curtains or break the rear door locks of the vehicles while parked or when drivers are resting. Otherwise, drivers can be corrupted or threatened. Moreover, criminals use technologies such as jammers to obscure the Global Positioning Systems (GPS) of the trucks and disable their localisations (Transport Security Manager 2, personal communication, February 16, 2022). Cargo theft occurring during secondary distribution is more opportunistic in nature. The preferred method is usually to steal the cargo from the delivery truck while it is parked or left unattended during a delivery. In some instances, thieves follow the delivery trucks and steal the products immediately after they have been delivered (Transport Security Manager 1, personal communication, February 25, 2022; Police Officer 2, personal communication, February 18, 2022).

A new recent trend in the illicit supply of medicines, especially high value ones, is to offer sample products to potential customers. In these cases, criminals break into the facilities where medicines are stored and take some pictures of the available products. These photos can then be immediately sent to the client or broker for confirmation or remain as a portfolio to be shown to new potential customers. Criminals may also grab one or two packs of medicines as samples (Pharmaceutical Security Manager 2, personal communication, February 22, 2022; Pharmaceutical Security Manager 3, personal communication, February 25, 2022).

#### Robbery

Robberies are less common than burglaries or thefts and occur mainly during transport. In hospitals or other physical facilities, both the threat and use of violence are usually not planned, but rather stem from the unexpected presence or reaction of either employees or security staff. Robberies against cargos include thefts from moving vehicles or hijacking via a forced stop. The first method is carried out on highways usually by three or more cars, one of which approaches the truck while the others block the highway (Transport Security Manager 2, personal communication, February 16, 2022). Generally, criminals jump from their own car onto the sunroof of the loaded truck, cut off the seal, and remove the goods. Another method used to rob cargos is hijacking, which involves the forced stoppage of trucks parked in unsecured areas via force, violence, or threats against drivers. The whole vehicle is then stolen or unloaded.

#### Fraud

Another method employed by organised criminals in the theft of medicines is by fraud, which occurs primarily through deception as criminals masquerade as legitimate transport operators. While fraudsters ordinarily target cargos, they can also focus on hospitals and warehouses. The companies who outsource their transportation are especially vulnerable because they rely on the spot market and online freight auction sites. Therefore, criminals deliberately focus their activities on these key areas, often exploiting vulnerabilities and gaps within companies’ due diligence checks or subcontracting procedures. Criminals utilise a diverse array of techniques, ranging from either presenting themselves as an entirely fictitious transport company, directly assuming the identity of a legitimate trading company, or providing false delivery instructions to drivers and companies. This latter technique is also known as driver call divergence, which is when drivers or companies are deceived into delivering to a different destination than the one intended (TT CLUB et al., 2021). Another type of fraud is when legitimate businesses operate with fraudulent intentions. While the company appears to be trustworthy, in fact, it may have been established or infiltrated by a criminal organisation (Transport Security Manager 2, personal communication, February 16, 2022). Furthermore, deceptive methods are also being used to stop vehicles without the use of violence or force. Criminals may also impersonate police officers and pretend to carry out routine road checks. In doing so, they stop the truck and take over it. Similarly, thieves can fake accidents by blocking the road, and as soon as the driver steps out of the truck, they get on board and leave the scene.

### Post-conditions: Transportation and Storage

Stolen medicines and medical devices always require transportation after the commission of thefts. In most instances, these products are transported and stored in illegal private warehouses belonging to either the leading actors of criminal organisations or the stealing units themselves (Prosecutor, personal communication, February 23, 2022). Their location is either nearby the setting of the theft or based in those areas where the territorial influence of the criminals is greatest. These storage facilities usually do not provide adequate temperature or other requirements causing medicines to lose their effectiveness or even become dangerous for patients (Di Giorgio & Russo, 2019).

After being stored in these warehouses, the stolen products do not necessarily need to be further transported covertly. This is because they are often laundered while stored in the warehouses, meaning that by the time they leave the premises they are already reintroduced into the legal pharmaceutical supply chain and ready to be sold via legitimate channels. In these instances, there is no type of transnational movement either after the theft or prior to the sale of the illicit medicines.

On the contrary, if the stolen products need to be concealed while transported across borders, criminal organisations have to decide which type of transportation method is most suitable, depending primarily on the quantity of the goods. In the case of a large number of medicines or medical devices, criminals corrupt conniving couriers for this specific task and generally use the same mode of transportation used for other illicit trafficking. Conversely, in the case of small quantities of pharmaceutical products, the methods used to move them are either via postal express services or ant-smuggling. On the one hand, criminals use legitimate and well-known delivery companies to send products inside regular parcels, which go largely undetected. On the other, small quantities of products are hidden within the personal luggage of unsuspecting people travelling by airplane, train, or other modes of transport (Tribunale di Cremona, 2018). These smugglers are often females or elderly people that, if stopped as part of security checks, can justify their travel by arguing that they are bringing back medicines that are not available in their own country for personal use or for the treatment of one of their relatives (Police Officer 1, personal communication, February 7, 2022). In all cases, criminals may rely on already established smuggling routes and channels (e.g. drug trafficking, counterfeits).

Overall, since clients usually want to be able to verify the medicines they are receiving, in the case of transnational movement, it is often important that there is a common language between the origin and destination countries of the stolen products. As the packaging of medicines is country-specific, medicines destined for one country are preferably stolen in countries that either speak the same language or in which the specific product has the same name (Pharmaceutical Security Manager 3, personal communication, February 25, 2022).

### Post-conditions: Selling

The stolen products can be sold by reintroducing them into the legal market or by trading them in the illegal market. The selling channels via the legal market often entail large-scale or transnational movements, as opposed to small-scale and local illicit businesses. However, criminals are increasingly using social networking platforms and e-pharmacies as selling channels due to the COVID-19 pandemic, which has led to an ever-growing pool of clients looking for medical products online.

#### Legal Market

Thanks to the support of conniving professionals or bogus companies, criminals produce deceptive documents referring to fake transactions that, in turn, allow them to sell stolen medicines in the legal supply chain to unwitting parallel trade wholesalers or national wholesalers, buyers on the secondary market, or legitimate pharmacies or businesses (e.g. convenience stores) (Pharmaceutical Security Manager 1, personal communication, February 9, 2022, Police Officer 1, personal communication, February 7, 2022; Prosecutor, personal communication, February 23, 2022). Reintroducing medicines or devices into the legal market is preferred by criminals when dealing with large quantities of stolen products. For example, a group of Italian individuals allegedly established an articulated network of conniving or fake wholesaler companies and pharmacies to launder hospital prescription medicines stolen from logistics operators, in order to resell them to legitimate wholesalers in other EU markets. According to the prosecutors, some shell companies were created in the UK and Ireland to provide false documentation, while other legitimate businesses either managed by the same fences or by conniving professionals were used to generate real or fake transactions for the stolen medicines. As a consequence of this scheme, this group was able to launder stolen medicines with an estimated value of €10 million in only a few months between 2011 and 2012 (Tribunale di Monza, ﻿^2015^).

#### Black Market

Online channels, street markets, and conniving legitimate businesses (e.g. gyms and sex shops) are the primary channels used by criminals to sell their stolen products on the black market. They also often rely on conniving professionals in healthcare and other sectors that operate as brokers who engage with the final clients (Police Officer 1, personal communication, February 7, 2022). Online channels include the deep web, but also social networking platforms and marketplaces or unauthorised online pharmacies, which are proper online websites that look legitimate to most consumers, who are often unaware of the illicit source of the products (Parallel Trader, personal communication, February 15, 2022; Pharmaceutical Security Manager 2, personal communication, February 22, 2022). In some countries, it is illegal to sell medicines online, but the lack of awareness among the population helps this selling channel to spread (Health Regulatory Authority 1, personal communication, February 22, 2022). Criminals may actively engage patients who look for prescription medicines in online forums and offer and sell the stolen products to them. An example of this case was noticed in Italy by AIFA in relation to the sale on the black market of medicines used for the treatment of hepatitis C (Di Giorgio, 2022). Finally, conniving legitimate businesses pertain to those instances in which the owners take advantage of their occupation or position to establish illicit sales among their clients to generate additional profits.

### Post-conditions: Clients

The final clients for stolen medicines and medical devices are approached either in the legal or illicit market depending on the type of stolen products and the purposes of the clients themselves.

#### Witting Clients

Clients that intentionally purchase stolen products are attracted by either the competitive prices of these products or their unavailability in their local markets. Different types of witting clients can be identified. The first set includes private doctors, other professionals in the healthcare system (e.g. healthcare managers or procurement executives), or owners of pharmacies and convenience stores that decide to illegally purchase medicines or medical devices because of the low prices (Health Regulatory Authority 1, personal communication, February 22, 2022). Due to their role and sectorial networking, they may either get offered the stolen products by conniving professionals or are already on the lookout for illicit products themselves and know how to approach criminals via their networks (Police Officer 1, personal communication, February 7, 2022). In some cases, these clients have only suspicions regarding the illicit origin of the products, but the economic benefits of the purchase prevent them from investigating further. This may also connect to a lack of awareness about the potential damage that these products cause to patients.

The second type of witting clients are individuals who access the black market because they cannot legally afford high-priced pharmaceutical products and look for a more economical alternative for their treatment. Another reason is that these products are unavailable on the legal market, not only because of shortages but also because these treatments are not marketed at all in their country. Therefore, individuals—particularly wealthy ones—opt for the black market to purchase these medicines (Pharmaceutical Security Manager 1, personal communication, February 9, 2022, Parallel Trader, personal communication, February 15, 2022). The third type of witting individual clients regards those that illegally purchase lifestyle medicines for recreational activities or enhancing their performance (e.g. sport- or sex-related). Both the illicit purchase and subsequent use of these drugs are often highly normalised within the environments and social groups frequented by these individuals.

#### Unwitting Clients

When stolen products are reintroduced into the legal supply chain, many clients are wholly unaware that the products they are purchasing are of unlawful origin. These clients comprise either hospitals and healthcare facilities, private professionals, wholesalers, and parallel importers/distributors, or even single patients (Pharmaceutical Security Manager 1, personal communication, February 9, 2022). These clients purchase illicit medicines on the legal market from ostensibly reliable sources (e.g. wholesalers or local pharmacies conniving with criminals).

#### Criminal Clients

Finally, another minor destination for stolen products concerns other criminals that use them for alternative illicit purposes. One of these involves the stealing of pharmaceutical waste or brand-new products to refurnish counterfeiters with input materials or information to create adulterated or fake medicines. Other examples are either networks of drug dealers who use stolen medicines as alternatives for other illicit drugs or criminals in the sporting world who use doping substances to alter regular or illicit competitions.

## Conclusion

This paper highlights a typology of criminal schemes used to steal and re-sell pharmaceutical products and summarises their main common traits. The analysis testifies that organised theft of medicines comprises a wide range of different criminal actions that are often transnational in nature. This crime requires the involvement of several actors with distinct roles or competencies and the participation of conniving legitimate professionals or companies. Despite this complexity, the comparative analysis of the identified criminal schemes and of the collected case studies demonstrates that stable and structured organised crime groups are only marginally involved in this crime. They act predominantly as facilitators of some specific activities (e.g. the movement or concealment of stolen products), especially in those territories where their presence and connections are higher. On the contrary, the main actors are independent criminals who create joint ventures or collaborate in loose networks according to transient criminal opportunities. This trend corresponds to the one observed in other illicit markets, such as illicit tobacco (Di Nicola & Terenghi, 2016), counterfeiting (Lavorgna & Sergi, 2014), illegal puppy trade (Maher & Wyatt, 2021), or wildlife trafficking (Moreto & Van Uhm, 2021).

The variety of actors involved and the consequent multiplicity of potential cooperations and *modi operandi* pose a significant challenge for law enforcement agencies and regulatory authorities in identifying illegal behaviours or anticipating potential threats. This complexity is further aggravated by the dual nature of the pharmaceutical market, in which a licit and an illicit dimension coexist (Savona et al., 2017). The analysis denotes, on the one hand, the central role played by legitimate professionals or companies that operate both on the licit and illicit side of the market exploiting their expertise and business connections to favour different stages of the theft of medicines. On the other hand, it highlights how differences in regulations and management of legal pharmaceutical production and distribution across countries can influence the demand for illicit goods and create opportunities for legitimate products to be diverted into the criminal market. Passas (1999) defines these transnational differences as “criminogenic asymmetries”: inequalities among states in their economic, regulatory, or social structures that enable or enhance criminal behaviours by increasing the demand for illegal products, incentivising illegal practices, and hindering effective controls.

Regarding the theft of medicines in the EU, one example of these asymmetries is the pricing policies and reimbursement regimes for pharmaceutical products adopted by different states. While most countries at least partially regulate prices in the pharmaceutical market to ensure affordable access to pharmaceutical products (OECD, 2008), decisions about the pricing and reimbursement policies for medicines are a national competence of EU MS (Vogler et al., 2018). Existing differences in the medicines that come under the scope of the national policies can enhance the illicit demand for certain pharmaceutical products. Additionally, reimbursement regimes can also influence the illegal demand for medicines. The lack of affordable products and low or limited reimbursement creates an incentive to acquire illicit pharmaceuticals at lower prices (Espìn & Rovira, 2007).

Similarly, price regulation can also impact the availability of certain products due to the marketing decisions of pharmaceutical industries. For example, external price referencing is the most common approach adopted by EU MS, which means that prices are set according to the benchmark prices for the same or similar medicines in comparable countries (Toumi et al., 2014). This entails that the prices set in one country directly influence the prices in other countries, thus potentially either leading to unaffordable medicines or causing fewer or delayed launches of new drugs in lower-income countries (OECD, 2008). The fact that some products are not available in some markets and distributed in others generates a growing demand for these products in the first countries that could be fulfilled by criminals diverting legitimate products from the latter ones.

Moreover, regulatory asymmetries do also affect the capacity of authorities to control potential criminal behaviours. For example, in several EU MS, the procurement and tendering for buying pharmaceutical products are decentralised to regional authorities or even to individual hospitals (Vogler et al., 2018). These local entities may lack the capacity to thoroughly scrutinise all the offers they receive or verify the legitimacy of the actors involved. Additionally, they often do not have access to communication and alerts from international or foreign authorities. As a result, this increases the opportunities for criminals to infiltrate the legal market with illicit or stolen product.

Therefore, effective efforts to combat this type of crime require continuous attention to new or emerging criminal *modi operandi* and addressing existing asymmetries through coordinated actions among public and private stakeholders across different countries and jurisdictions. This is especially important given criminals’ adaptability to regulatory changes, counteracting strategies, and market developments, such as the emergence of specific products in response to the COVID-19 pandemic (TAPA, 2021). To achieve this goal, it is fundamental to increase both the sharing of information and the level of cooperation between all the relevant actors at both the national and transnational level. This conclusion has been supported by most of the interviewed stakeholders. Several issues can be identified in the current ways information about thefts of medicines is generated and communicated. First, the reporting and investigation of thefts of medicines are typically managed locally without involving other authorities or stakeholders. This approach has a negative impact on the efforts to combat this crime, as medicines stolen in one country may be traded in the legal or illegal market of another country. In this regard, Italian authorities have committed to periodically publishing and disseminating a list of stolen or under short-supply medicines to national and foreign stakeholders (Di Giorgio, 2022; Di Giorgio & Russo, 2019). However, this positive example is currently an exception (Di Giorgio et al., 2019a, 2019b). Second, systematic information on the theft of medicines is still lacking and available data are often inconsistent across countries. For instance, many cases of diversion or losses recorded along the pharmaceutical supply chain may conceal thefts and fraud. This implies that the number of criminal events could be significantly higher than the officially reported levels. Factors such as the lack of a shared definition of this crime type, low awareness by public authorities and private actors, and the absence of effective reporting systems contribute to the underestimation and underreporting of medicine thefts. Moreover, some logistics operators may be reluctant to share data on actual stolen or missing products to protect their brand, which makes public disclosure of this event rare. For example, if a single operator were to report thefts frequently, its customers would probably switch their business to competitors. However, if all stakeholders acknowledge the extent of this issue, it could reduce the incentive for not reporting criminal events. Finally, there is a need to increase awareness among potential clients about the consequences of buying stolen products. This could lead unwitting clients to increase their knowledge about this crime and to exercise more formal or informal controls before making an unwise purchase. Similarly, it could prompt conscious clients to consider the potential harm to their health or that of their patients from using illicit products.

Given the current lack of information on this crime, the results and conclusions of the extant study should be considered cautiously and in line with its explorative nature. Additionally, it is important to note that this analysis primarily focuses on EU MS, and while some vulnerabilities in the pharmaceutical sector may be common worldwide and similar criminal behaviours have been documented in other countries (Bate et al., 2010; Chávez, 2021; Marchione et al., 2020; PSI, 2021), further research is needed to generalise some of the emerging findings. Nevertheless, this analysis emphasises the importance of dedicating more attention to this crime which is, or may become, a serious issue in many countries worldwide given its deleterious impact on national economies and the health of citizens. This attention is further required also considering the growing demand for medical products due to population ageing and changes in clinical procedures (OECD, 2021), the ever-increasing commercial value of the pharmaceutical industry, and the limited access to certain medicines that may generate new opportunities for criminals (Council of Europe, 2021; Interpol, 2020).

## Supplementary Information

Below is the link to the electronic supplementary material.

Supplementary file1 (DOCX 41 KB)

## Data Availability

Data sharing not applicable to this article as no datasets were generated or analysed during the current study. Information on interviews and case studies analysed are provided in the Appendix.

## Footnotes

1 […] (a) the manufacturing, the keeping in stock for supply, importing, exporting, supplying, offering to supply or placing on the market of: (i) medicinal products without authorisation where such authorisation is required under the domestic law of the Party; or (ii) medical devices without being in compliance with the conformity requirements, where such conformity is required under the domestic law of the Party; (b) the commercial use of original documents outside their intended use within the legal medical product supply chain, as specified by the domestic law of the Party (Council of Europe, 2011, p. 5).

2 “Pharmaceutical crime involves the manufacture, trade and distribution of fake, stolen or illicit medicines and medical devices. It encompasses the counterfeiting and falsification of medical products, their packaging and associated documentation, as well as theft, fraud, illicit diversion, smuggling, trafficking, and money laundering”(Interpol, 2013).

3 The diversion includes any case of legitimate pharmaceutical products that are approved and intended for sale in one market being illegally moved and sold in another market (Zabyelina & Noguera, 2022).

4 This collection used the Nexis® Data Lab/Metabase, which is the largest repository of international, national, and local sources, and queries were conducted in twenty-four languages focusing on European sources to identify articles containing the words “Theft”, “Robbery”, or “Burglary” in association with “Medicine”, “Pharmaceuticals”, or “Medical devices”. The 1177 records identified were further scrutinised in order to identify a set of 87 relevant unique cases of the theft of medicines that could be used to inform the classification of the most common *modi operandi* of criminals.

5 More details about the characteristics of these seven criminal schemes are described in Dugato & Sidoti (2022)
