# Supplementary material for: The Organised Theft of Medicines: a Study of the Methods for Stealing and Reselling Medicines and Medical Devices in the EU and Beyond
Source: Eur J Crim Pol Res. 2023 May 31:1–22. Online ahead of print. doi: 10.1007/s10610-023-09546-w (PMC10231288; doi:10.1007/s10610-023-09546-w)
Supplement: Supplementary file 1 — Supplementary file1 (DOCX 41 KB) [file 10610_2023_9546_MOESM1_ESM.docx]

Appendix

Table 1. Details on the interviews, including the role of the respondents, their countries and the dates of the interviews

| **Role** | **Countries** | **Date of Interview** |
| --- | --- | --- |
| Police Officer 1 | Italy | 07/02/2022 |
| Police Officer 2 | Netherlands | 18/02/2022 |
| Hospital Pharmacists | Italy | 15/02/2022 |
| Transport Security Manager 1 | Italy | 25/02/2022 |
| Transport Security Manager 2 | Belgium | 16/02/2022 |
| Pharmaceutical Security Manager 1 | Italy | 09/02/2022 |
| Pharmaceutical Security Manager 2 | Switzerland | 22/02/2022 |
| Pharmaceutical Security Manager 3 | Germany | 25/02/2022 |
| Pharmaceutical Security Manager 4 | Italy | 25/02/2022 |
| Pharmaceutical Security Manager 5 | France | 04/03/2022 |
| Health Regulatory Authority 1 | Serbia | 22/02/2022 |
| Health Regulatory Authority 2 | Netherlands | 12/03/2022 |
| Parallel Trader | Belgium | 15/02/2022 |
| Prosecutor | Italy | 23/02/2022 |
| Pharmaceutical association | Sweden | 08/03/2022 |

Table 2. Details on the analysed judicial cases

| **Judicial Cases** | **Year of the operation** | **Countries where the theft occurred** | **Other Countries Involved** |
| --- | --- | --- | --- |
| Case Study 1 | 2014/2015 | Italy | Cyprus, Czech Republic, Finland, Germany, Hungary, Latvia, the Netherlands, Romania, Slovakia, Slovenia, Spain, and the UK |
| Case Study 2 | 2014 | Italy | Albania, Greece, Latvia, Hungary, Romania, Slovenia, and Slovakia. |
| Case Study 3 | 2012 | Italy | Denmark, Germany, Ireland, the Netherlands, Spain, Sweden, and the UK |
| Case Study 4 | 2017 | Germany | Colombia, Italy, Spain, and the United States |
| Case Study 5 | 2018 | Italy | France, Germany, Egypt, Saudi Arabia, and Syria |
| Case Study 6 | 2018 | Greece | Cyprus, Germany, and Italy |

Table 3. Countries mentioned in the 87 relevant cases of theft of medicines retrieved from news and press releases between 2015 and 2022

| **Mentioned Countries** | **Number of Mentions** |
| --- | --- |
| Italy | 37 |
| Egypt | 12 |
| Germany | 10 |
| Spain | 7 |
| The UK | 6 |
| Bulgaria | 5 |
| Czech Republic | 5 |
| Greece | 5 |
| Russia | 4 |
| The Netherlands | 4 |
| Tunisia | 4 |
| Sweden | 3 |
| Ukraine | 3 |
| France | 2 |
| Morocco | 2 |
| Saudi Arabia | 2 |
| Albania | 1 |
| Portugal | 1 |
| United Arab Emirates | 1 |
| Yemen | 1 |
